# Efficacy of Case-Based Learning in Anatomy

**DOI:** 10.7759/cureus.20472

**Published:** 2021-12-16

**Authors:** Muralidhar Reddy Sangam, Praveen K, Vinay G, Raju R Bokan, Roonmoni Deka, Amandeep Kaur

**Affiliations:** 1 Anatomy, All India Institute of Medical Sciences, Guwahati, IND

**Keywords:** competency based medical education, teaching learning method, medical education, anatomy, case-based learning

## Abstract

Introduction

In the present Competency-Based Medical Education (CBME), learning is more student-centered where the students take the responsibility for their learning. Anatomy is an important basic science that lays the foundation for clinical courses in the Bachelor of Medicine and Bachelor of Surgery (MBBS) curriculum. To make it interesting and clinically useful, several innovative teaching-learning methods like case-based learning (CBL) and problem-based learning (PBL) are introduced. The present study was taken up to know the effectiveness of CBL as a teaching-learning method in Anatomy in improving the knowledge and retention of acquired knowledge.

Material and Methods

This was an interventional cross-over study carried out at NRI Medical College and General Hospital, Guntur, Andhra Pradesh. Two hundred students studying in first-year MBBS were included in the study and divided into two batches. The batches - A and B - were exposed to CBL and didactic lecture, respectively, in the first month for Topic I, and then cross-over was done in the second month for Topic II. The knowledge of the students before and after the sessions was assessed by pre-session and post-session multiple-choice question (MCQ) tests. Knowledge retention was assessed by another MCQ test conducted four weeks after the post-session test.

Results

The average difference of the scores between pre-session and post-session tests in the CBL group for Topics I and II (4.01±1.17 and 3.8±1.6) are significantly more compared to the didactic lecture method (3.3±1.3 and 1.9±1.2). The average difference of the scores between the post-session tests and retention-tests in the CBL group (0.122±1.05 and 0.18±1.04) were further compared to the lecture method (0.016±0.95 and 0.09±0.8) for Topics I and II, respectively. There was a significant increase in the proportion of students with scores above 50% in the post-session test and retention test in the CBL group compared to the didactic lecture group.

Conclusion

Results from the pre-session tests, post-session tests, and retention tests for both the topics indicate that CBL as a teaching-learning method in Anatomy is a more effective method for improving and retention of knowledge.

## Introduction

Background

In the present competency-based medical education (CBME), there are guidelines for teaching and learning methods so that Indian medical graduates can be lifelong learners. The curriculum has been restructured from discipline-based to competency-driven. There is a shift from traditional didactic lectures to small group teaching to make learning more student-centered, where the learners become responsible for their learning. To promote active and effective interactive learning, various innovative approaches like case-based learning (CBL), problem-based learning (PBL), and team-based learning (TBL) are adopted in undergraduate medical education [1]. CBL promotes active learning by utilizing clinical case scenarios which reflect real-life experiences that students will face during the clinical phase of their medical education [2]. CBL is structured so that trainees explore clinically relevant topics using open-ended questions with well-defined goals [3]. Cases are written as problems with the history, physical findings, and laboratory results of a patient. By discussing a clinical case related to the topic taught, students evaluate their own understanding of the concept using higher-order cognition. This process encourages active learning and gives a more productive outcome [4]. CBL has several advantages like promoting self-directed life-long learning, introducing basic medical sciences in a coherent manner closely related to topics in clinical sciences, and reinforcing the reasoning, collaborative and communication skills of students [5,6].

Aim

In undergraduate medical education, a clear understanding of Anatomy and its application is important, for a strong foundation in clinical practice. Anatomy is perceived to be a difficult and dry subject by the majority of students. To make it interesting and clinically useful, a good understanding of the anatomical basis of diseases is required. One of the important innovative methods being used to facilitate this is CBL. The aim of this study is to find out the efficacy of CBL in learning Anatomy among first-year MBBS students.

Objectives

The first objective of this study is to compare the effectiveness of CBL as a learning method in Anatomy among first-year MBBS students with the traditional lecture method using pre-test and post-test scores. Second, we want to compare the effectiveness of CBL with the traditional lecture method in knowledge retention among first-year MBBS students using post-test and retention test scores.

This article was presented as a scientific poster in Poster Session as a part of Advanced Course in Medical Education at St. John's Medical College, Bengaluru, Karnataka, India on 12-11-2021.

## Materials and methods

This interventional cross-over study was carried out in the Department of Anatomy, NRI Medical College and General Hospital, Chinakakani, Guntur from November 2020 to August 2021. Permission was obtained from Institutional Ethics Committee, NRI Medical College and General Hospital (No. IEC/NRIMC&GH/241/2020). The study included 200 students studying first MBBS after getting their written consent to participate. Students absent for any session or test were excluded from the study.

Learning material for the students was prepared for two topics in Gross Anatomy by constructing specific case-based scenarios with four or five analytical questions for each. This material was reviewed by the senior faculty and appropriate changes were made after discussion. The faculty was trained in CBL with the help of the Medical Education Unit, NRI Medical College and General Hospital. The 200 students were divided into two groups of 100 each according to their registration numbers. Group A included students with registration numbers 1 to 100 and group B with 101 to 200. In the first month, after the dissection in Topic I (Axilla), group A followed CBL and group B followed the traditional lecture. The students were assessed with pre-session and post-session multiple-choice question (MCQ) test scores. To assess the concept retention, another MCQ test was conducted after four weeks. Each test included 10 MCQ questions, which are validated. In the second month, after the dissection in Topic II (Front of Thigh), group A followed the didactic lecture and group B followed CBL. The students were assessed with pre-session and post-session MCQ test scores. Another MCQ test was conducted four weeks after to assess the concept retention. In CBL, 100 students were divided into five groups of 20 each. Each group was guided by a tutor acting as a facilitator. The students are asked to go through the case scenarios, discuss in the group, and answer the questions using the textbooks and reference books. Access to the internet was provided to the students for resource material. The role of the tutor was to guide the discussion and summarize the topic towards the end of the session.

**Table 1:** Study design CBL: case-based learning

Topic I (Axilla)	Pre-session test	Group A CBL	Group B Didactic lecture	Post-session test	Knowledge retention test (four weeks after post-session test)
CROSS					
Topic II (Front of thigh)	Pre-session test	Group A Didactic lecture	Group B CBL	Post-session test	Knowledge retention test (four weeks after post-session test)

Statistical analysis

Continuous data is represented as Mean ± SD. Parametric tests were used for analysis as the data followed normal distribution. The comparison test of significance used was independent t-test. Chi-square test was used to compare the percentage of students above and below 50% scores in all the tests. The level of significance was taken at 5% level. SPSS for Windows, Version 16.0 (Released 2007; SPSS Inc., Chicago) was used.

## Results

Two hundred students studying first-year MBBS participated in the study. Of these, 30 were excluded from the study as they are absent for one or more sessions/tests. Of the 170 students included, 106 are females and 64 are males.

The average difference between pre-session and post-session MCQ test scores in the CBL method for Topics I and II is 4.01±1.17 and 3.8±1.6, respectively, which is better compared to the scores in the lecture method (3.3±1.3 and 1.9±1.2) and the difference is statistically significant (Table 2). Knowledge retention scores (difference between post-session test and retention test scores) are more in the CBL method (0.122±1.05 and 0.18±1.04) compared to the lecture method (0.016±0.95 and 0.09±0.8) for Topics I and II, respectively (Table 3). There was no significant increase in retention test scores in the CBL group when compared to the lecture group for Topic I but for Topic II the difference was significant. However, the mean score of the retention test in the CBL group is more when compared to another group. There was a significant increase in the number of students with above 50% score in post-session tests and retention tests for both the topics in the CBL group when compared to the lecture method group (Tables 4-5).

**Table 2:** Comparison of average difference between pre-session test and post-session test scores in the two groups. CBL: case-based learning

Topic	Session	Group	Average difference (V) mean±SD	P-value
Topic I	CBL	A	4.01±1.17	0.001*
	Lecture	B	3.3±1.3	
Topic II	CBL	B	3.8±1.6	0.0001*
	Lecture	A	1.9±1.2	

**Table 3:** Comparison of average difference between post-session test and retention test scores in the two groups. CBL: case-based learning

Topic	Session	Group	Average difference (V) mean±SD	P – value
Topic I	CBL	A	0.122±1.05	0.391
	Lecture	B	0.016±0.95	
Topic II	CBL	B	0.18±1.04	0.05*
	Lecture	A	0.09±0.8	

**Table 4:** Comparison of percentage of students with scores above and below 50% for Topic I (Axilla). CBL: case-based learning

Test	Performance	Group A (CBL)	Group B (Lecture)	P-value
Pre-session test	Below 50%	86	97	0.01*
	Above 50%	14	3	
Post-session test	Below 50%	15	36	0.001*
	Above 50%	85	64	
Retention test	Below 50%	17	38	0.01*
	Above 50%	83	62	

**Table 5:** Comparison of percentage of students with scores above and below 50% for Topic II (Front of Thigh). CBL: case-based learning

Test	Performance	Group A (Lecture)	Group B (CBL)	P value
Pre-session test	Below 50%	83	96	0.005*
	Above 50%	17	4	
Post-session test	Below 50%	43	22	0.003*
	Above 50%	57	78	
Retention test	Below 50%	43	29	0.01*
	Above 50%	57	71	

## Discussion

As per the Vision 2015 document of the Medical Council of India (MCI), emphasis should be on the introduction of case scenarios for classroom discussion. CBME guidelines by National Medical Commission (NMC) adopted in 2019 requires a shift from didactic lectures to small group teaching method and give early clinical exposure to students to make Indian medical graduates more competent and skilled [7]. Learners need to see the relevance of the study and engage themselves actively in the topic discussed. By realizing the importance of including the early clinical exposure, horizontal and vertical integration was introduced where the clinical relevance of the topic is discussed. Researchers in basic science courses have concluded that CBL stimulates the students to apply cognitive skills as per clinical context [8] and develops and improves problem-solving skills, thus emphasizing the pathophysiological basis in clinical context [9,10]. CBL provides better opportunities to students for the usage of different resource materials and better interaction with their peers and instructor [11]. CBL promotes deeper learning; that is, learning that goes beyond simple identification of correct answers and is more aligned with either evidence of critical thinking or changes in behavior and generalizability of learning to new cases [9].

A number of studies have been done to assess the effectiveness of CBL compared to the lecture method. Few concluded to have better performance with CBL [12-14], few with lecture method [15,16], and few with no difference between two methods [17]. They described that these variations may be due to the implementation of the CBL session and the size of the group.

In the present study, the mean difference between pre-session and post-session test scores is significantly higher in the group following CBL compared to the other group following the lecture method, for both the topics. This indicates that CBL is an effective teaching-learning method helping the students to build on prior knowledge, integrate the knowledge, and consider an application to future situations. Similar observations were reported by Shaifaly et al. [18] and Nair et al. [19] in Anatomy, and Singhal et al. [20] and Blewett et al. [21] in Microbiology. The mean difference between post-session test and retention test scores is higher in the CBL group. This explains that retention of acquired knowledge is more in the group following CBL as it encourages the student to develop self-directed deep learning. A similar comparison was made by Chamberlain et al. [22] and Ciraj et al. [23] and reported that retention of knowledge is better in the CBL group.

Understanding the advantages of CBL, the option of introducing it in learning Anatomy in the existing curriculum can be considered. This requires understanding the needs of assessment, teamwork (by the curriculum committee, medical education unit, and individual department), and faculty sensitization program in CBL. This should be followed by identification of the topic in which CBL can be implemented and defining specific learning objectives for them. Feedback from different stakeholders can improve the entire process.

Limitations

The study was done in a single institute and with one batch of students. Only two topics were selected for the study. The enthusiasm of the students towards a new teaching-learning method may have played a role in the inclination of the results to CBL and hence the impact on long-term change in behavior is to be identified.

## Conclusions

To achieve the goals of the Indian medical graduate, it is hard to believe that a single method of teaching-learning can achieve it. CBL is an effective teaching-learning method that may be used in combination with didactic lectures allowing the students to practice real-world clinical cases and can promote learning capabilities enhancing the academic experience of medical students. This method also enhanced pre-class preparation, student’s understanding, student-teacher interaction, communication skills, self-directed learning, and knowledge absorption. 
